# Extreme Prematurity: A Case Report on the Importance of Multidisciplinary Consultations Before and After Maternity Ward Discharge

**DOI:** 10.7759/cureus.76518

**Published:** 2024-12-28

**Authors:** Oana-Alexandra Peta, Alexandru Dinulescu, Ana Prejmereanu, Oana Maria Petrescu, Mirela Luminita Pavelescu

**Affiliations:** 1 Pediatrics, Carol Davila University of Medicine and Pharmacy, Bucharest, ROU; 2 Pediatrics, Children's Clinical Hospital Dr. Victor Gomoiu, Bucharest, ROU; 3 Pediatrics, Emergency Hospital for Children "Grigore Alexandrescu", Bucharest, ROU; 4 Pediatric Cardiology, Clinical Hospital of Obstetrics and Gynecology Prof. Dr. Panait Sîrbu, Bucharest, ROU

**Keywords:** extremely preterm infant, high-risk pregnancy, motor development disorder due to prematurity, retinopathy of prematurity, severe left ventricular hypertrophy

## Abstract

Extreme prematurity involves a series of complications that a multidisciplinary team should manage. Taking into account the risks related to premature newborns, such as maternal-fetal infections, intrauterine growth restriction, and certain comorbidities associated with young gestational age, our objective is to highlight the importance of a multidisciplinary team in approaching cases with an unfavorable prognosis. This is a case report of an extremely preterm newborn who came from a high-risk pregnancy and needed long-term hospitalization in the Neonatal Intensive Care Unit (NICU) and mechanical ventilation.

## Introduction

The extreme prematurity pathway is littered with an abundance of risks and complications, including neurodevelopment impairments, anemia, bronchopulmonary dysplasia, retinopathy of prematurity, congenital heart disease, and prolonged jaundice [1-3].

In Romania, there is high mortality due to delivery before 28 weeks of gestation [2]. Preterm infants represent a challenge not only for neonatologists but also for the entire health system and the parents. They need multidisciplinary follow-up, are economically consumptive, and have a psychological impact on the parents [4-6].

Preterm infants face various deficiencies and comorbidities, demanding comprehensive medical care during their first weeks of life. Almost without exception, extreme preterm neonates are respiratory support dependent, with a high risk of desaturation, prompting medical specialists to opt for intubation and mechanical ventilation [7-9].

Moreover, healthcare providers should raise awareness of what prenatal follow-up is concerned about. In this way, prevention exceeds the value of treatment and plays an important role in reducing preterm birth and complications. Although the exact etiology of preterm labor is not completely understood, some theories suggest that intrauterine infections and inflammation during pregnancy are the most common causes, accompanied by unhealthy lifestyles, fertility treatments, medical or mental disorders, and substance addiction [10].

## Case presentation

We report the case of an extremely preterm female newborn at 25.6 gestational age, with a very low birth weight of approximately 730 g, extracted by cesarean section from a pelvic presentation at a Grade III maternity hospital in Bucharest, Romania. The newborn came from a high-risk pregnancy, with intrauterine growth restriction; her mother was affected by hypothyroidism, gestational hypertension, SARS-CoV-2 infection, Candida infection, and Group B Streptococcus (GBS) positive during pregnancy. Physical examination of the neonate included a modified general condition, skin with generalized cyanosis, and shallow breaths for which alveolar recruitment was performed in the Neopuff system in the delivery room. The skin became pink with acrocyanosis and she had rhythmic heart sounds, no murmurs, diminished tone and reactivity, absent archaic reflexes, clinically undetectable congenital malformations, and normotensive anterior fontanelle. Because the newborn had significant cyanosis and generalized hypotonia, she obtained an Apgar score of 4 at 1 minute, 7 at 5 minutes, and a score of 8 at 10 minutes, having an unfavorable prognosis.

Immediately after birth, she received a course of two antibiotics encompassing Meropenem IV (14 days) in association with Amikacin IV (5 days) in neonatal doses, according to the age of gestation. Prophylaxis of apnea attacks was commenced with caffeine citrate in the loading dose of 20 mg/kg and then maintenance doses up to 35 weeks of gestation (corrected age). The patient underwent inotropic support with IV dopamine (2.5 mg/kg/min) and continuous intravenous therapy for hypotension (BP = 48/19/28 mmHg).

In light of these facts, the newborn was admitted to the Neonatal Intensive Care Unit and was placed at the thermal neutral point. It must be noted that alveolar recruitment in the NeoPuff system (Fisher & Paykel Healthcare, Auckland, NZ) was continued for 20 minutes, with the patient maintaining oxygen saturation (SpO2) in the first 10 minutes of life according to resuscitation guidelines. A slow bolus of sodium chloride 0.9% NaCl, hydroelectrolyte balancing, and intake infusion were administered. It was decided to perform a chest X-ray, which highlighted the appearance of bilateral matte glazing. Moreover, blood gas analysis revealed respiratory acidosis, thus our team decided that the patient entailed intubation and mechanical ventilation. She received a Curosurf (Chiesi USA, Inc., Cary, NC, US) dosage of 200 mg/kg. Then, mechanical ventilation was started in the high-frequency oscillatory ventilation (HFOV) system with synchronized intermittent mandatory ventilation with sedation with midazolam if necessary. Considering the extreme prematurity and the serious general condition of the newborn, our team started the treatment for the prophylaxis of bronchopulmonary dysplasia with hydrocortisone hemisuccinate, according to national and international guidelines, for two weeks.

Prophylaxis of hemorrhagic disease of the newborn was performed every week with intramuscular doses of phytomenadione. Enteral nutrition was initiated on the third day of life via oral gavage with the formula quantity gradually increased according to digestive tolerance. Neonatal screening cultures were collected. The peripheral cultures were sterile and the blood culture was negative at five days. At 36 hours of life, biological samples were collected (blood count, C-reactive protein (CRP), aspartate transaminase (AST), alanine aminotransferase (ALT), urea, creatinine, lactate dehydrogenase (LDH)). The blood count showed significant leukopenia with neutropenia and thrombocytopenia, with an absent inflammatory biological syndrome (Table 1), corroborated with the patient's serious general condition, led us to opt for a treatment escalation plan comprising of Vancomycin IV (for 14 days), Klacid IV (for 7 days) and human immunoglobulin IV (1 g/kgc/day for 3 days) in neonatal doses, according to gestational age. Taking into account the broad-spectrum antibiotic regimen and the presence of the central venous catheter, from day 7 of life, the prophylaxis of fungal infections with IV Fluconazole was performed in neonatal doses.

**Table 1:** Blood test results at 36 hours of life CRP - C-reactive protein; AST - Aspartate transaminase; ALT - Alanine transaminase; LDH - Lactate dehydrogenase

Blood tests	Patient values	Normal values
White blood cell (10^3^/mm^3^)	3.78 ↓	8.16-14.56
Neutrophils (10^3^/μL^3^)	0.74 ↓	1.73-6.75
Lymphocytes (10^3^/μL^3^)	1.73 ↓	1.75-8
Hemoglobin (g/dl)	15.6	13.4-20
Platelets (10^3^/mm^3^)	85 ↓	144-449
CRP (mg/L)	3.94	0-5
AST (U/L)	8.92 ↓	15-60
ALT (U/L)	8 ↓	13-70
LDH (U/L)	626 ↑	160-450
Urea (mg/dl)	32.58	11-36
Creatinine (mg/dl)	0.9	0.3-1.2

**Figure 1:**
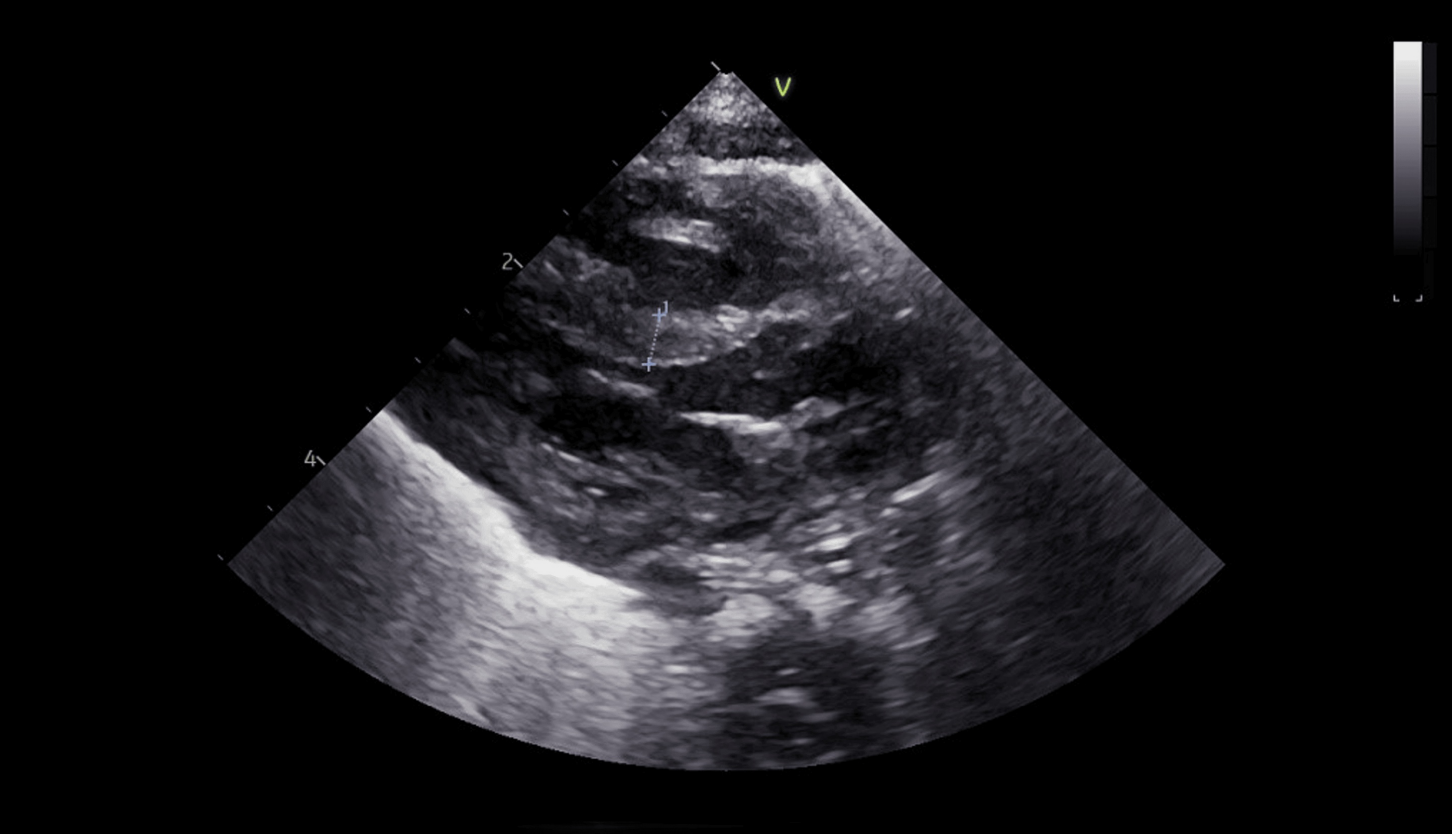
Echocardiography: parasternal long-axis view showing left ventricle hypertrophy

**Figure 2:**
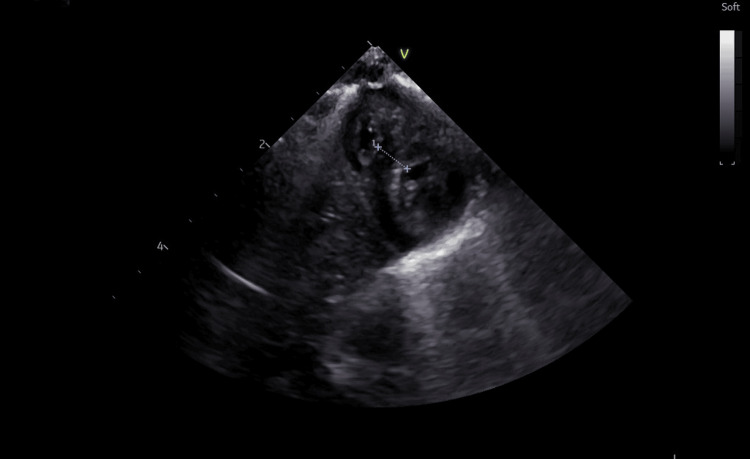
Echocardiography: short-axis view of the left ventricle showing left ventricle hypertrophy

**Figure 3:**
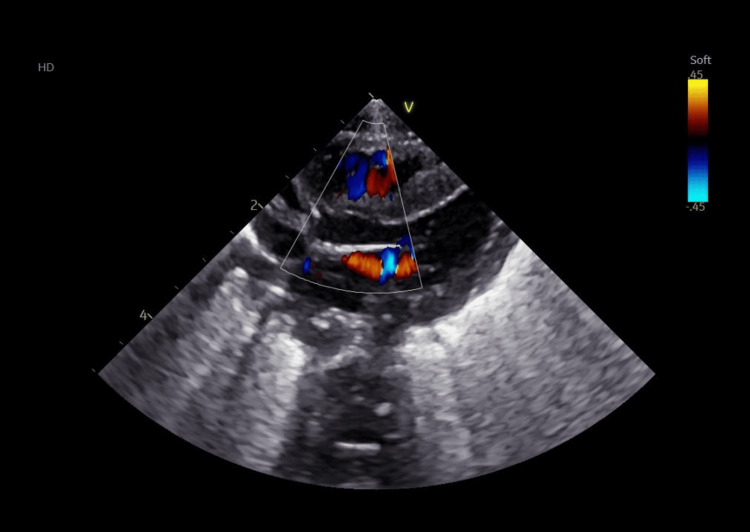
Echocardiography: Color Doppler of the aortic outflow tract showing no tract obstruction

Furthermore, a series of investigations was conducted. Transfontanellar ultrasound revealed bilateral grade II intraventricular hemorrhage with mild ventricular dilation. In addition, echocardiography visualized severe left ventricular hypertrophy, without signs of obstruction (Figures 1-3), therefore Propranolol was added to the therapeutic scheme.

Several attempts were made to switch mechanical ventilation from the HFOV system to the synchronized intermittent mandatory ventilation (SIMV) system depending on the respiratory parameters, the general condition of the newborn, and the blood gas analysis. Then, taking into account the clinical and paraclinical condition of the newborn, extubation was performed and non-invasive continuous positive airway pressure (CPAP) ventilation continued for another 3 days, with the transition to oxygen therapy under a cephalic tent for another 29 days and then in free flow for another 35 days.

On the 18th day of life, the patient presented several desaturations of up to 60% and purulent secretions were aspirated on the endotracheal tube (ETT). Clinical examination described a distended, non-depressible abdomen, so a thoraco-abdominal X-ray was performed. It emphasized the presence of atelectasis of the right middle lobe, hyperinflated lung, dilated intestinal loops, and an edematous intestinal wall. The abdominal ultrasound exhibited fecal impactation and a large amount of gastric residue. Our team decided to change antibiotic therapy with Imipenem/Cilastatin IV (for 14 days) and Colistin IV (for 7 days) in neonatal doses. One week later, extubation was practiced, with the patient placed under non-invasive CPAP ventilation, with a slow transition to oxygen tent therapy, considering the favorable evolution of the patient.

To detect premature retinopathy, we requested an ophthalmological consultation, which highlighted the presence of plus factor, arteriovenous sounds at the posterior zone II vascular ends. An intravitreal injection with Avastin was recommended and a thorough follow-up. To perform the procedure, sedation with midazolam and fentanyl was administered, and the infant presented multiple desaturations and bradycardia, which responded to balloon ventilation and mask for 1 minute; then during the procedure, SpO2 of 98% and heart rate (HR) of 160 bpm was maintained. At the end of the procedure, desaturation was up to SpO2 of 80% with spontaneous return. Our team decided to intubate the patient and ensured that ventilation was initiated in the SIMV system. However, the infant presented multiple desaturations up to SpO2 of 70%, so we were forced to switch mechanical ventilation to the HFOV system and perform a chest X-ray that revealed the appearance of bilateral matte glazing. Based on the guidelines, Curosurf 200 mg/kg per dose and antibiotic treatment with Tazocin IV in neonatal doses (for 7 days) were administered, with favorable outcomes under treatment.

Not long after this episode, systematized clonus of the lower limbs was observed during a clinical examination. We took into account a neurological condition due to extreme prematurity. Seizures were managed by administering phenobarbital in neonatal doses and the patient was referred to a pediatric neurologist. The neurological evaluation concluded that a feasible diagnosis was motor development disorder due to extreme prematurity and recommended physical therapy and periodic follow-up.

During hospitalization, the newborn presented jaundice that required three sessions of phototherapy with eye protection. She received the Bacillus Calmette-Guérin (BCG) and Hepatitis B virus (HBV) vaccines, and the Guthrie test was performed to rule out metabolic disorders. We tested the infant for cystic fibrosis and spinal amyotrophy, according to the informed agreement signed by the relatives. The control analyses performed revealed the blood count to be within normal limits for age, without biological inflammatory syndrome, and neonatal screening cultures without clinical significance for the child (Klebsiella pneumoniae extended-spectrum beta-lactamase (ESBL) positive).

At the time of discharge, she remained afebrile and displayed a satisfactory general condition, a pale pink complexion, and a reducible umbilical hernia. She was stable both cardiologically and pulmologically and fulfilled her nutritional needs, with good tonus and reactivity.

**Figure 4:**
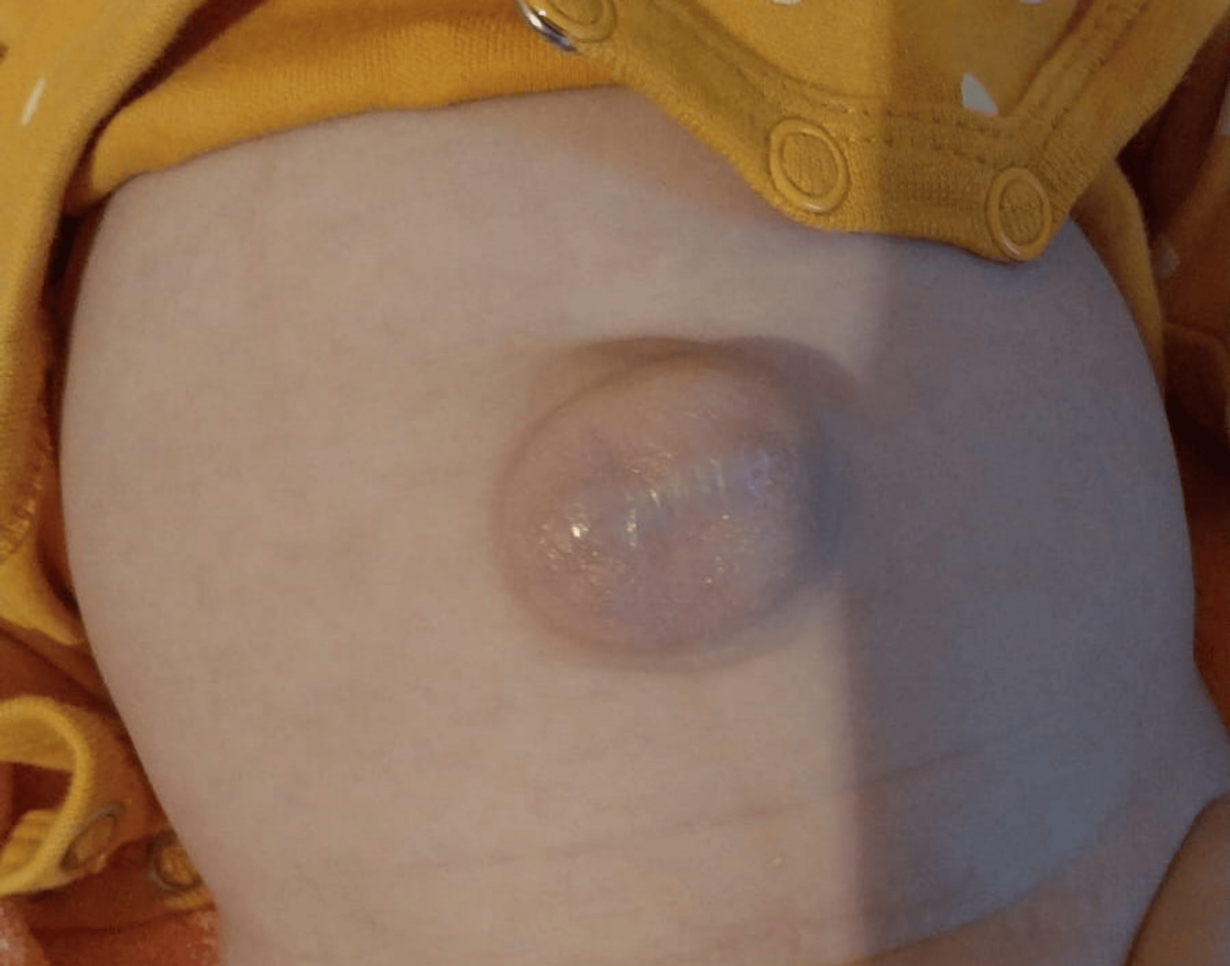
Umbilical hernia

**Figure 5:**
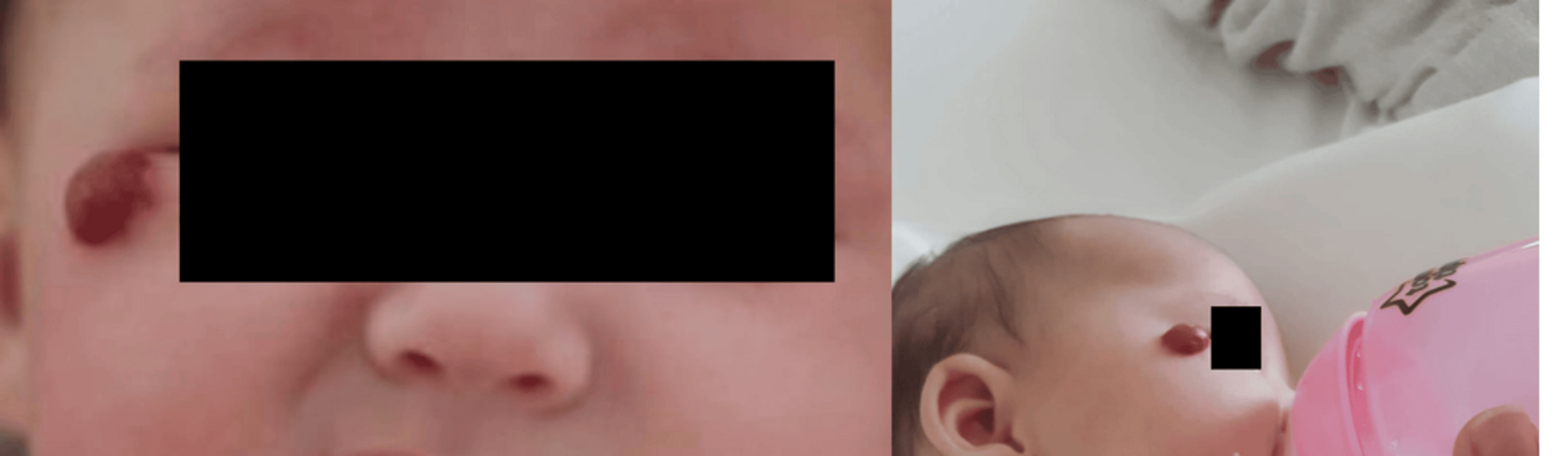
Hemangioma under the left lower eyelid

Taking into account extreme prematurity and long-term hospitalization in the NICU, hearing retests in the audiology clinic, ophthalmology and neurological consultations, and kinesiotherapy were recommended after discharge from the maternity ward. At the follow-up consultations, the infant presented a favorable evolution, ascending weight curve, and cardiorespiratory balance despite chronic lung disease without respiratory infections, an umbilical hernia (Figure 4), and hemangioma under the left lower eyelid (Figure 5); therefore, we recommended a consultation with pediatric surgery and to repeat the transfontanellar and abdominal ultrasound.

## Discussion

Efforts are being made to decrease the incidence of prematurity through prevention. This pregnancy was partially monitored, with the patient coming from a low socio-economic environment along with a complex obstetric pathology. Prematurity is defined as a birth that occurs before 37 complete weeks (less than 259 days) of gestation. It is associated with considerable risk of morbidity and mortality, particularly among extremely preterm infants (i.e., gestational age <28 weeks) [11,12]. To prevent complications of prematurity, antenatal corticosteroids and antibiotic therapy were administered and delivery was delayed as long as the medical situation allowed. There was a good obstetrician-neonatologist collaboration. Over two dozen randomized trials have confirmed that a course of antenatal corticosteroid therapy (ACS) administered to patients at risk for preterm birth reduced the incidence and severity of respiratory distress syndrome (RDS) and mortality in their offspring [13].

The extremely premature newborn came from a pregnancy with an infectious risk: SARS‑CoV‑2 infection occurring a few days before delivery, and the cervical culture was positive for GBS, which predisposed to premature birth. SARS-CoV-2 plays a significant role in inducing vascular and circulatory pathologies during fetal life [14,15]. The preterm neonate presented clinical and paraclinical signs of infection from the first hours of life, making it challenging for the neonatologist to administer antibiotic therapy to reduce the risk of neonatal morbidity and mortality. Sepsis continues to be challenging to diagnose due to its non-specific clinical signs and symptoms, emphasizing the importance of early detection [16,17]. Another challenge in this case was the prevention of fungal infections, as the premature newborn was treated with antibiotics and hospitalized for a long time in intensive care. Candida auris poses a serious threat to infection control and patient care since it can produce invasive infections with a high fatality rate. It has been linked to outbreaks in hospital environments and is typically resistant to several antifungal medications [18]. Nystatin is used prophylaxis (birth weight less than 1500 g), orally, 3 times a day for 6 weeks in neonatal intensive care unit (with a greater than 10% rate of invasive candidiasis) [19].

Alveolar recruitment was carried out using the Neopuff system in the delivery room, and the newborn later required mechanical ventilation. The medical team was attentive to both the needs of the newborn and the anticipation and prevention of acute and chronic complications. Mechanical ventilation (MV) is a lifesaving intervention, but it also risks injury to the lungs, brain, and other organ systems. Supporting gas exchange while minimizing harm is the key therapeutic goal and challenge of MV in neonates [20-22]. Oxygen supplementation is an important component of neonatal intensive care for the preterm infant. Ideally, oxygen administration provides adequate oxygenation for metabolic needs while avoiding the consequences of both hypoxemia and hyperoxia [23-25].

During hospitalization in the neonatal intensive care unit, the newborn was also evaluated from a cardiological point of view. This category of newborns presents a risk for patent ductus arteriosus. Periodic evaluation led to the diagnosis of severe left ventricular hypertrophy, without signs of obstruction; the clinical and paraclinical evolution under treatment was favorable. Patent ductus arteriosus (PDA) occurs commonly in preterm infants, especially in those with respiratory distress syndrome [26-28]. We also consider the fact that this newborn comes from a mother with hypothyroidism, which can lead to neonatal heart disease. Maternal hypothyroidism (MH) progeny have increased susceptibility to both acquired cardiovascular disease (CVD) in adulthood and congenital heart disease [29-31]. There is a high risk of developing atherosclerotic cardiovascular disease, some examples include premature birth, endocrine disorders, and chronic kidney disease [32,33].

Extremely premature infants are very precious, having a difficult and winding journey in neonatal intensive care. Their survival is due not only to the efforts of the neonatology team but also the multidisciplinary team and, in equal measure, the care of the parents after discharge. Parents were instructed to recognize possible complications and types of crying (pain, regurgitation, hunger, lower abdominal discomfort, and fatigue) at home, so there was a close collaboration between the neonatologist, pediatrician, and family doctor, and the child's evolution after discharge was favorable. Several methods were reported in the scientific literature for the classification of the infant's cries to automatically detect the need behind their tears and help the parents and caretakers [34].

This category of preterm neonates is at increased risk of pediatric hospitalization in the first year of life, being prone to respiratory infections, feeding difficulties, and weight gain. In our country, great efforts have been made for immunization against respiratory syncytial virus, and we can now vaccinate all high-risk newborns. The premature infant presented above was immunized since maternity, with the parents continuing the scheme through the family doctor. There is a study that addressed the main causes and limitations of neonatal readmission and how they can be prevented [35]. The parents of the premature infant complied with the National System vaccination scheme, as these infants need protection against multiple viruses and bacteria, especially after the COVID-19 pandemic [36,37].

The infant presented with infantile hemangioma in a high-risk area, the right lower eyelid, after discharge from the maternity ward. Low birth weight (LBW), prematurity, female sex, multiple gestations, and family history of infantile hemangioma (IH) are some of the statistically proven risk factors for developing IH [38].

## Conclusions

To save extremely premature neonates, the effort of a maternity medical team is not enough and a multidisciplinary team and responsible and trained parents are needed to prevent mortality and morbidity. Once the preterm infant manages to overcome the acute complications and leaves intensive care, we must consider the chronic complications of prematurity. After discharge, the premature newborn should be enrolled in a follow-up program to prevent or minimize developmental delay through the early identification of risk factors and referral to appropriate treatment programs.
